# Clinical dental finding in Iranian horses

**DOI:** 10.1002/vms3.329

**Published:** 2020-07-31

**Authors:** Lotfollahzadeh Samad, Hamid Tavanaeimanesh, Hossein Mehr Azin, Seyyed Hosein Moadab, Ali Reza Vajhi

**Affiliations:** ^1^ Department of Internal Medicine Faculty of Veterinary Medicine University of Tehran Tehran Iran; ^2^ Department of Surgery and Large Animal Internal Medicine Faculty of Veterinary Medicine, Science and Research Branch Azad University Tehran Iran; ^3^ Department of Clinical Science Faculty of Veterinary Medicine Razi University Kermanshah Iran; ^4^ Department of Surgery and Radiology Faculty of Veterinary Medicine University of Tehran Tehran Iran

**Keywords:** age, dental diseases, horse, management, prevalence

## Abstract

**Background:**

A horse's well‐being is directly related to the management of its dental health. A good knowledge of the epidemiology and aetiology of dental disorders could help the owners and clinicians to prevent not only dental problems but also severe gastrointestinal diseases.

**Objectives:**

In this study we report the prevalence of dental disorders in horses in Iran.

**Methods:**

We examined 317 horses randomly in eight provinces in Iran and 21 diseases were characterized in the examined horses. The observed diseases were compared among different breeds, genders and ages of the examined horses.

**Results:**

The factor of age among the other three factors was more important in the incidence of diseases because most of the diseases found were significantly different among age groups. Between different breeds examined, only cheek teeth cemental caries in Kurdish and Arabian horses was significantly different (*p* = .022). Enamel point with an occurrence of 34.4% was the most common disease. Broken cheek teeth were more prevalent in male horses in comparison with female horses (*p* = .035).

**Conclusion:**

Our study showed a moderate prevalence of dental disorders in Iranian horse clubs, which could be reduced with better management.

## Graphical abstract

This study was done on a big population of horses to find the most common dental disorders and find their relation to important factors.

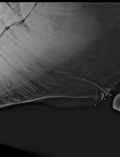

## 1 INTRODUCTION

The horse industry is becoming a popular field for investment in Iran, and the horse population has grown rapidly in the last decade. The knowledge of owners about the welfare of their horses especially in relation to dental care has improved over these years. Routine dental care is necessary for every horse and dental problems can have a direct effect on horse welfare.

The majority of oral problems in horses are related to dental diseases, and routine oral cavity examination should be carried out in equine practice. Equine practitioners generally spend up to 10% of their time on oral cavity–related issues (Deans, 1965). In the United States dental disorders are the third‐most common disorder in large animal practice (Traub‐Dargatz, Salman, & Voss, 1991).

**FIGURE 1:**
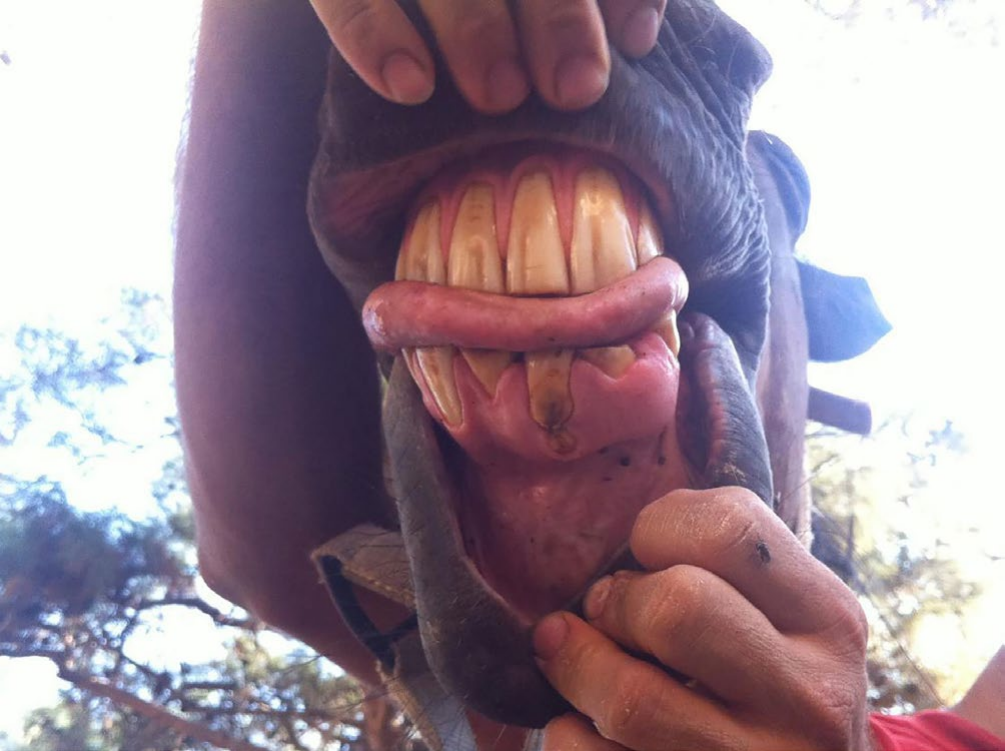
This horse suffered from abnormally shaped mandibulary incisors that are additionally suffering focal enamel caries – a rare finding at this site caused by developmental defects

Some dental problems are as a result of developmental problems (Figure 1) like overjet and underjet. The most common equine dental problem is the overgrowth of the buccal edge of the maxillary cheek teeth (CT) and lingual edge of the mandibular CT. Tooth sharpness, named ‘enamel points’, can lacerate the cheek or tongue soft tissues and causes a disorder in the chewing process (Masey, Keen, & Dumbell, 2010).

A common sign of periodontal diseases and dental caries is halitosis (Dixon et al., 2000). Weight loss is less commonly caused by dental problems because horses with these disorders spend more time on chewing food. ‘Diastema’, which defines a gap between the CT, is a predisposing condition for periodontal diseases (Easley, 2009).

The aim of this study was to perform a cross‐sectional analysis of equine dental disorders in Iran according to age, breed and gender groups. Our hypothesis was that clinically diagnosed problems and findings may have a relationship with our groups. Results of this study could help owners and veterinarians to know the prevalence of dental problems between different groups of horses so they can pay more attention to susceptible groups.

## 2 MATERIALS AND METHODS

### 2.1 Animals

Eight provinces of Iran were visited by ambulatory veterinary surgeons from September 2014 till September 2016. The horses monitored belonged to studs and jumping clubs. At first, the history of horses was taken from the owners and recorded in special forms. In the anamnesis information such as age, gender, breed, any history of previous colic, the presence of long strands of forage and undigested whole‐grain particle in the faeces, quidding of food, halitosis and trauma to the skull and face were recorded. Also, the behaviour of horse when he or she was gagged was noted.

After taking the history of the horses all were sedated with detomidine (0.01 mg/kg IV) and mouth was opened by using a McPherson speculum. The oral cavity was observed by using head light and mirror, and then all the teeth were palpated thoroughly and X‐ray was taken later, as it was necessary for accurate diagnosis.

The head was also examined from cranial and lateral aspect for any asymmetrical signs or signs of trauma. Any secretion from nostrils or halitosis was noted and recorded. Frontal and maxillary sinuses were percussed in order to detect any sign of sinusitis. Tongue was examined for the presence of any possible trauma or inflammation.

Cases are subdivided into one of four age groups: <5 years (group 1), 5–10 years (group 2), 11–15 years (group 3) and >15 years (group 4). We put our horses into five different breed groups, all show jumping breeds were categorized in one group because their management was the same, the other breeds were Thoroughbred, Arabian, Turkman and Kurdish. An examination chart including 21 of the most common equine dental disorders was prepared.

The results obtained in this study were analyzed by SPSS version 16.0 software. Descriptive statistical methods and the chi‐square test were used for the analysis of the data. The test confidence level was 95%.

## 3 RESULTS

A total of 317 horses were examined including 166 females and 151 males. Among the studied horses, there were 130 Thoroughbred, 72 Show jumping, 43 Arabian horses, 42 Turkman and 30 Kurdish horses.

In this study the horses were evaluated for 21 dental disorders and it was found that 69.4% of examined population show at least one of these disorders. 26.5% of horses had just one disorder, 18% showed 2 and 15.8% 3 disorders and 5.4%, 2.5%, 0.6% and 0.6% of horses showed 4, 5, 6 and 7 simultaneous diseases respectively.

Cheek teeth cemental caries was the only disorder that was different between breeds and the difference was seen in Kurdish and Arabian horses (*p* = .022), there was not any statistical difference in the different disorders between the other breeds.

Fractured teeth were more prevalent in male group as compared with the female group (*p* = .03).

**FIGURE 2:**
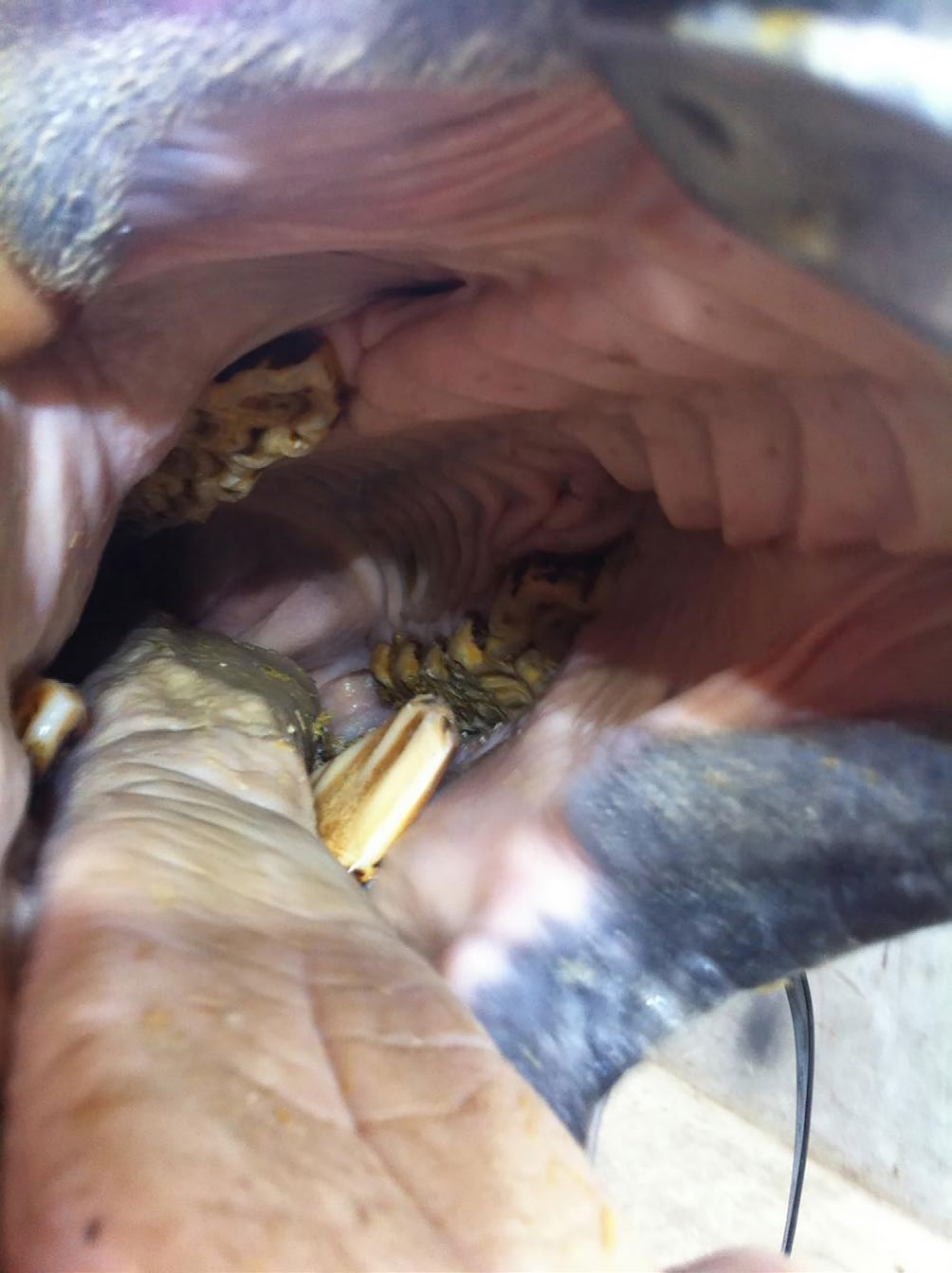
Long hook at 306 subsequent to fractured 206

**FIGURE 3:**
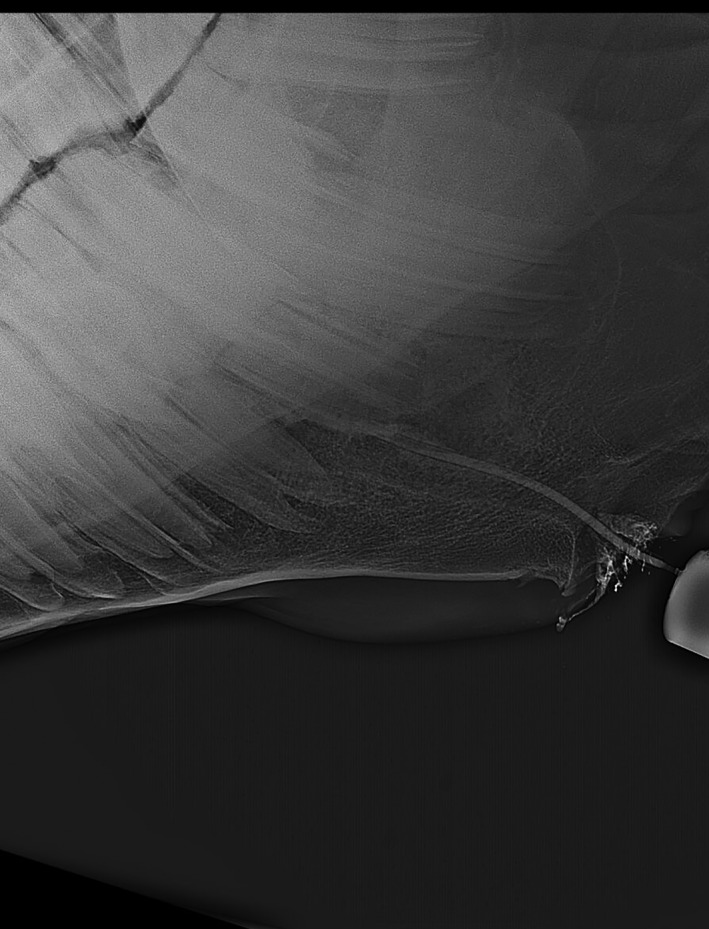
Apical infection in 410 which has fistula, X‐ray has taken by contrast media to show the direction of fistula

Most dental diseases found in the examined horses in this study showed a significant statistical difference in age groups with the exception of the following diseases: diastema, tongue laceration, periodontal diseases, apical infection (Figures 2 and 3), overjet and underjet, which were not statistically different between age groups (Table 1).

**TABLE 1:** Distribution of absolute and relative (%) frequency in dental disease and disorders in different age groups

Age disorders	Group 1 <5 years (72 horses)	Group 2 5–10 years (114 horses)	Group 3 11–15 years (83 horses)	Group 4 >15 years (48 horses)		
	Number Per cent	Number Per cent	Number Per cent	Number Per cent	Total	*p*‐value
Enamel point	16 22.2% AB	42 36.8% A	38 45.8% BC	13 27.1% C	109 34.4%	.012
Supernumerary teeth	4 5.6% A	1 0.9%	0 0 A	0 0	5 1.6%	.02
Wave mouth	1 1.4% AB	5 4.4% C	9 10.8% A	11 22.9% BC	26 8.2%	.0005
Step mouth	0 0 AB	0 0 DC	19 22.9% AD	14 29.2% BC	33 10.4%	.0005
Hook	6 8.3% ABC	24 21.1% A	26 31.3% B	13 27.1% C	69 21.8%	.05
Cheek teeth cemental carries	0 0 ABC	15 13.2% ADE	23 27.7% BD	14 29.2% CE	52 16.4%	.0005
Incisor teeth cemental carries	1 1.4% A	4 3.5%	6 7.2%	6 12.5% A	17 5.4%	.039
Cheek teeth diastema	2 2.8%	7 6.1%	11 13.3%	4 8.3%	24 7.6%	.087
Incisor teeth diastema	2 2.8%	0 0	0 0	0 0	2 0.6%	.08
Dislocated teeth	5 6.9%	4 3.5% A	7 8.4%	8 16.7% A	24 7.6%	.04
Broken cheek teeth	0 0	0 0 A	1 1.2%	3 6.2% A	4 1.3%	.008
Broken incisor teeth	7 9.7% AB	5 4.4%	0 0 B	0 0 A	12 3.8%	.007
Cheek ulcer	0 0 ABC	19 16.7% A	16 19.3% B	9 18.8% C	44 13.9%	.002
Lingual ulcer	0 0	0 0	0 0	0 0	0 0	0
Cheek teeth periodontal diseases	6 8.3%	10 8.8%	9 10.8%	9 18.8%	34 10.7%	.25
Incisor teeth periodontal diseases	3 4.2%	4 3.5%	5 6%	4 8.3%	16 5%	.6
Apical infection	2 2.8%	0 0	2 2.4%	0 0	4 1.3%	.23
Overjet	3 4.2%	8 7.2%	12 14.5%	4 8.3%	27 8.5%	.09
Underjet	0 0	0 0	0 0	0 0	0 0	0
Cheek cap retention	4 5.6% AB	0 0 A	0 0 B	0 0	4 1.3%	.003
Incisor cap retention	3 4.2% A	0 0 A	0 0	0 0	3 0.9%	.02

Note

Similar letters indicate significant difference (*p* < .05) between the specified *groups.

There was a statistical difference between horses in group 1 and group 2 in enamel point occurrence. Also a difference was shown between group 1 and group 3, but horses in group 4 did not show the same difference.

Eleven horses in group 4 and 9 horses in group 3 had wave mouth. Wave mouth was seen just in one case in the group 1, and there were statistical differences between these age groups.

Cheek teeth cemental carries was not seen in group 1, but in other groups it occurred and 15, 23 and 14 horses with CT cemental carries were seen, respectively, in groups 2–4.

We found at least one case from each disorder in our investigation with the exception of tongue ulcer and underjet. 69.4% of the examined horses had at least one disorder, among which 26.5% had just one problem and 0.6% had 7 problems simultaneously.

Enamel point was the most common problem in our study with a prevalence of 34.4%. The prevalence of this disorder in group 3 was 45.8% which was most prevalent among the age groups (Table 1).

**FIGURE 4:**
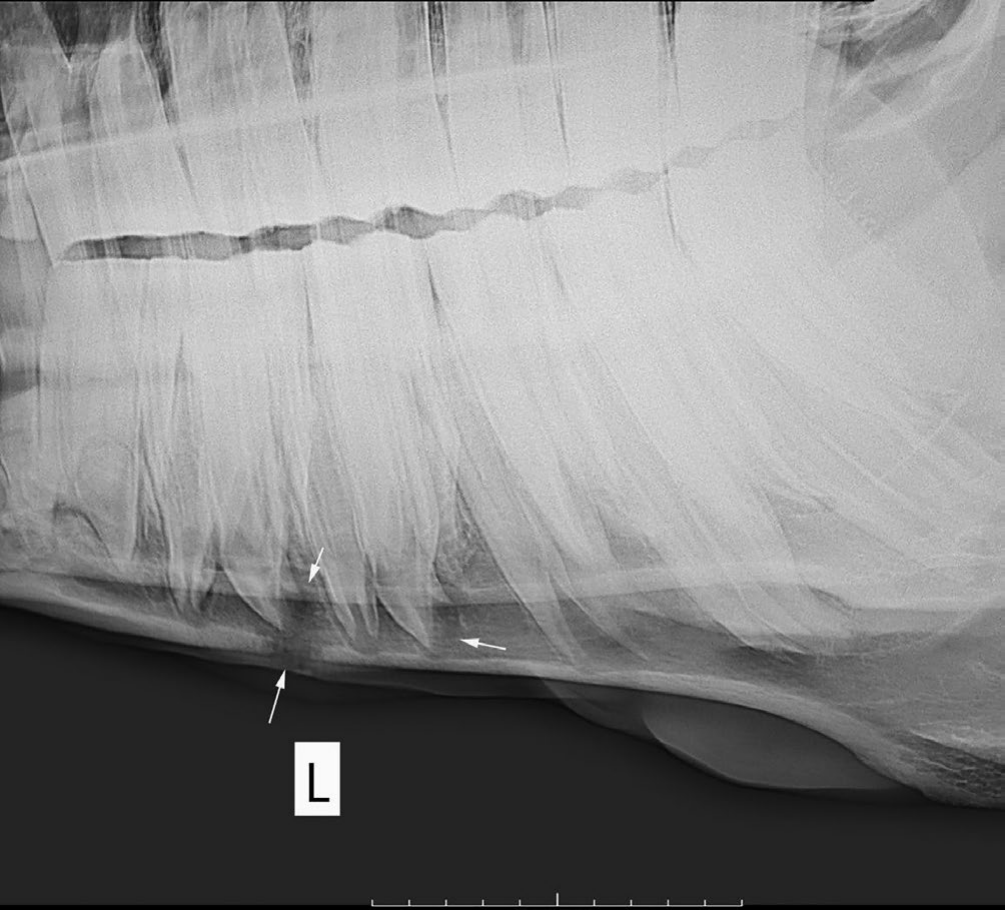
Apical infection in 307 and 308 which caused bone lysis

Dental hook (Figure 4) with 21.8% and CT cemental caries with 16.4% were recorded as the second‐ and three‐most common problems in our study (Table 1).

Cheek teeth periodontal disease was seen in 10.7% of our examined population and incisor teeth periodontal diseases were in 5% of horses in both cheek and incisor teeth horses aged >15 years showed a higher prevalence 18.8% and 8.3% respectively. No statistical difference was seen between age groups.

The content of feed rations was very similar between sites so we excluded it from our study. None of our horses had free grazing and the ration consisted of alfalfa and hay mostly in the ratio 3:1 and 0.2% BW concentrate and grain. Frequency of feeding horses in all the studs was three times a day.

## 4 DISCUSSION

In a study by Du Toit and her colleagues which was done on donkey's carcasses in 2008, the prevalence of dental disease was 80% (Du Toit, Burden, & Dixon, 2008). In another study it was found that only 4.5% of the examined horses never received any dental care during their life (Rodrigues, Dixon, & Bastos, San Roman, & Viegas, 2013).

In another study of 400 horses referred for dental problems, 345 horses had CT problems, incisor and canine tooth problems which were observed in 44 and 11 horses respectively (Dixon et al., 2000).

In our study, similar to Dixon et al., the prevalence of CT diseases was more common than incisor ones (Dixon et al., 1999). In another study, (Du Toit et al., 2008) the prevalence of dental diseases was 62% which is a little lower than in our report (69.4%). The prevalence of dental problems in another study was 73.1% and only 12.3% of the horses examined did not show dental problems. In this study the prevalence of dental problems in younger horses was 28% and in older horses was 34% (Du Toit, Burden, & Dixon, 2009). In our study 30.6% of examined horses had no dental problems; which may be due to better management and more attention to dental care by owner and riders and progress in the treatments and management techniques used by veterinarians.

Dixon and his colleagues reported that 345 horses of 400 referral horses had dental disorders (Dixon et al., 1999). The recorded dental problems included: 20 hooks in the mandible, 16 diastemata, 15 mandible eruption cysts, 4 maxillary eruption cysts and 10 supernumerary teeth.

In another survey (Brigham & Duncanson, 2000) of 50 horses, enamel points were seen in 26% of CT.

Maslauskas et al reported 60.7% enamel points in both maxillary and mandibular CT. They found that 11.7% of enamel points were in the lingual surface of mandibular CT, and 39% were placed in the buccal edge of maxillary CT (Maslauskas, Tulamo, McGowan, & Kučinskas, 2009).

In present study, in Iran enamel points were the most common dental problem with a prevalence of 34.4% and a significant difference has been observed in different age groups. The prevalence of this disorder was higher in group 3 compared with other groups, authors believed that this higher prevalence was the result of the less attention of some owners to this age group because most of these horses were retired from sport competition and were used for breeding so some owners had fewer requests for floating of these horses.

In our study buccal ulcer had high prevalence and its occurrence had a significant correlation with aging, similar to other studies (Bottegaro et al., 2012; Vlaminck et al., 2006). As in group 3 we saw 19.3% and in >15 years it was 18.8%.

Dental hook was observed in 21.8% of the horses and our finding was in agreement with the Du Toit study which hooks were mostly found in the maxillary (Du Toit et al., 2009).

In our study fractured teeth were more prevalent in the male group as compared with the female group which may be correlated with their more aggressive behaviour Some authors believe that periodontal diseases are the most important buccal cavity problem in horses (Du Toit et al., 2008; Rodrigues et al., 2013), in the present study after enamel point, hook and cemental caries, periodontal disease was the most prevalent disorder.

Periodontal disease in horses has a relationship with the occurrence of diastema, abnormalities of wear and abnormalities of dental eruption and its prevalence has a relation with age (Du Toit et al., 2008).

## 5 CONCLUSIONS

The results of this study provide a better view of the aetiology of dental disease and the level of dental care in the Iranian horse industry, and it could help to improve welfare of horses. If owners and veterinarians know the aetiology of diseases and susceptible groups to special disorders, prevalence would be easier. This study is the most comprehensive study in this subject which was done up to now in Iran. Our study showed a moderate prevalence of dental disorders in Iranian horse clubs, which could be reduced with better management.

## CONFLICT OF INTEREST

None of the authors of this study have a financial or personal relationship with other people or organizations that could inappropriately influence or bias the content of the study.

## AUTHOR CONTRIBUTION

**Hossein Mehr Azin:** Investigation. **Samad Lotfollahzadeh:** Conceptualization; Formal analysis; Project administration; Writing‐original draft. **Hamid Tavanaeimanesh:** Conceptualization; Formal analysis; Funding acquisition; Investigation; Methodology; Supervision; Visualization; Writing‐original draft; Writing‐review & editing. **Seyyed Hosein Moadab:** Conceptualization; Formal analysis; Funding acquisition; Investigation; Methodology; Visualization. **Alireza Vajhi:** Investigation.

### Peer Review

The peer review history for this article is available at https://publons.com/publon/10.1002/vms3.329.
